# Effectiveness of shear wave elastography for assessing major salivary gland involvement in ankylosing spondylitis

**DOI:** 10.1590/0100-3984.2024.0121

**Published:** 2025-03-11

**Authors:** Irfan Atik, Seda Atik, Enes Gul

**Affiliations:** 1 Faculty of Medicine, Sivas Cumhuriyet University, Sivas, Turkey

**Keywords:** Spondylitis, ankylosing/diagnostic imaging, Inflammation, Salivary glands/physiopathology, Ultrasonography/methods, Elasticity imaging techniques/methods., Espondilite anquilosante/diagnóstico por imagem, Inflamação, Glândulas salivares/fisiopatologia, Ultrassonografia/métodos, Técnicas de imagem por elasticidade/métodos.

## Abstract

**Objective:**

To use shear wave elastography (SWE) in the evaluation of salivary glands in patients with ankylosing spondylitis (AS) who present with sicca symptoms.

**Materials and Methods:**

This was a prospective controlled study of patients diagnosed with AS and exhibiting sicca symptoms (study group) and of healthy volunteers (control group). The levels of antinuclear, anti-Ro, and anti-La antibodies were determined in blood samples. In both groups, parotid and submandibular glands were evaluated by ultrasound and tissue stiffness was determined by SWE. Intraclass correlation coefficients were used in order to assess reliability. The differences between the two groups were assessed by statistical methods, and a ROC curve analysis was performed to determine the predictive values.

**Results:**

A total of 66 patients with AS and 71 healthy volunteers were included in the study. There were no significant differences between the groups in terms of age or sex (*p* > 0.05). The intraand inter-rater reliability of SWE were good for the parotid gland (0.81-0.85 and 0.80, respectively) and for the submandibular gland (0.85-0.88 and 0.80, respectively). Statistically significant differences were found. Tissue stiffness in the parotid and submandibular glands, as determined by SWE, was significantly greater in the study group than in the control group (*p* < 0.05).

**Conclusion:**

Although there was no histopathological correlation in the parotid and submandibular salivary glands of patients with AS and sicca symptoms compared with the healthy volunteers, quantitative measurements showed greater tissue stiffness in the former group. In patients with AS, SWE guides salivary gland biopsy, which is the gold standard for diagnosing Sjögren’s syndrome.

## Abstract (pt)

**Objetivo:**

Foi realizada avaliação, utilizando elastografia por onda de cisalhamento (EOC), da glândula salivar de pacientes com espondilite anquilosante (EA) que apresentavam sintomas de secura.

**Materiais e Métodos:**

Em pacientes diagnosticados com EA e exibindo sintomas de secura, os níveis de anticorpos antinucleares, anticorpos anti-Ro e anti-La no sangue foram investigados. As glândulas parótidas e submandibulares bilaterais foram avaliadas utilizando ultrassom em ambos os grupos de pacientes e controle, e a rigidez do tecido foi registrada usando EOC. Estimativas do coeficiente de correlação intraclasse foram usadas para avaliar a confiabilidade. A diferença entre os dois grupos foi avaliada por métodos estatísticos. A análise da curva ROC foi realizada para determinar os valores de predição.

**Resultados:**

Foram incluídos no estudo 66 pacientes com EA e 71 voluntários saudáveis. Não houve diferenças significativas em idade e sexo entre os grupos (*p* > 0,05). A confiabilidade intra-avaliadores e interavaliadores da EOC para a glândula parótida (0,81-0,85 e 0,80) e glândula submandibular (0,85-0,88 e 0,80) foi considerada boa. Diferenças estatisticamente significantes foram encontradas nas medidas da EOC da glândula parótida e submandibular em pacientes com EA, indicando aumento da rigidez no grupo de pacientes (*p* < 0,05).

**Conclusão:**

Embora não tenha havido correlação histopatológica nas glândulas salivares parótidas e submandibulares bilaterais de pacientes com EA com sintomas de secura em comparação ao grupo controle, as medições quantitativas mostraram aumento da dureza do tecido. O uso da EOC em pacientes com EA orienta a biópsia da glândula salivar, que é o padrão ouro para o diagnóstico de síndrome de Sjögren.

## INTRODUCTION

Sjögren’s syndrome (SS) is a chronic autoimmune disease that affects the lacrimal gland and salivary glands and is characterized by xerophthalmia and xerostomia. In addition, mucosal surfaces such as the respiratory tract, gastrointestinal tract, and vagina may be affected, resulting in a condition called sicca syndrome. Many systemic involvements may accompany SS. It can have various clinical presentations, from musculoskeletal system involvement to life-threatening organ damage, and can occur in isolation or together with other autoimmune diseases, being categorized as primary or secondary SS, respectively^**(1-3)**^.

As is known, secondary SS is often associated with autoimmune rheumatic diseases such as rheumatoid arthritis, systemic lupus erythematosus, polymyositis/dermatomyositis, and systemic sclerosis. One of the rheumatic diseases we encounter most frequently in clinical practice is ankylosing spondylitis (AS). Patients with AS may also have complaints similar to those of patients with sicca syndrome. However, the number of studies examining the coexistence of SS and sicca syndrome is limited, typically in the form of case reports. The mechanism of that concomitance remains unknown^**(4-8)**^.

The diagnosis of SS is based on the 2016 American College of Rheumatology/European League Against Rheumatism classification criteria^**(9)**^. According to those criteria, the diagnosis can be based on the presence of anti-Ro antibodies or the results of a minor salivary gland biopsy. However, given the inadequacy of anti-Ro antibody measurement in daily practice and the fact that salivary gland biopsy is an invasive, time-consuming procedure, different diagnostic methods have begun to be investigated^**(10)**^. Many studies have shown that salivary gland ultrasonography is valuable in the diagnosis of secondary SS in autoimmune rheumatic diseases such as systemic lupus erythematosus and rheumatoid arthritis. Tissue homogeneity and differences in echogenicity are evaluated with B-mode ultrasonography. However, homogeneity of the salivary gland or mild abnormalities do not exclude SS. It has been reported that shear wave elastography (SWE) can successfully demonstrate chronic inflammation in the salivary gland^**(10,11)**^.

Although it is a relatively new diagnostic method, SWE has recently come to be used more widely in many areas. Its ability to make quantitative measurements, to give user-independent results, and to be easily applied have been factors in testing its utility in many fields, including gastroenterology, rheumatology, and oncology^**(12-16)**^.

The objective of this study was to investigate the guiding role of SWE before salivary gland biopsy in patients with AS and sicca symptoms.

## MATERIALS AND METHODS

The study was approved by the Institutional Ethical Review Board of Sivas Cumhuriyet University, in the city of Sivas, Turkey, and was conducted in accordance with the principles outlined in the Declaration of Helsinki (Decision no. 2024-01/03). All participating patients gave written informed consent.

This was a prospective study involving patients who presented to the rheumatology outpatient clinic between April and September of 2024 and were diagnosed according to the SpondyloArthritis International Society criteria^**(17)**^. Patients with conditions that may cause sicca symptoms, such as diabetes mellitus and thyroid diseases, were excluded, as were those receiving radiotherapy to the head and neck region, those using anticholinergic drugs, those previously diagnosed with SS, those with rheumatic or autoimmune diseases associated with secondary SS, those with signs of active infection in the salivary glands, glandular homogeneity, ductal changes, or mass lesions, and those showing hypoechoic areas on B-mode ultrasonography. The exclusion criteria were intentionally broad in order to distinguish any pathology strongly involving the salivary glands and to contribute to the diagnosis with SWE findings in the early period when B-mode ultrasonography findings were not observed. The duration of the disease was counted from when patients first experienced inflammatory low back pain. We also recruited a control group of 71 healthy volunteers.

Patients were questioned about oral and ocular symptoms according to the revised international classification criteria for SS. For oral symptoms, they were asked if they had experienced dry mouth persisting for more than three months, a need to drink water when swallowing dry foods, and whether they had swelling in their salivary glands. Regarding ocular symptoms, they were asked if they had experienced irritating dry eyes persisting for more than three months, a sensation of grittiness or sand in the eyes, and if they used eye drops more than three times a day. Patients who answered affirmatively to at least one of these questions were included in the study. Their blood samples were screened for antinuclear, anti-Ro, and anti-La antibodies with the enzyme-linked immunosorbent assay method^**(18)**^. The control group consisted of volunteers without any illness or complaints related to sicca syndrome.

**Figure 1:**
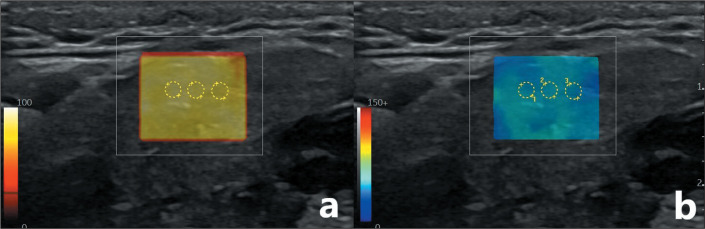
Evaluation of the submandibular gland by SWE in the transverse plane, showing a quality indicator (a) and measurement of tissue stiffness (in kPa) with three equal regions of interest (b).

All participants underwent evaluation with a real-time ultrasound system with elastography capability (Logiq E10s; GE HealthCare, Milwaukee, WI, USA), equipped with a 6-15 MHz linear transducer. The assessments were conducted by two radiologists who were blinded to the patient clinical data. Patients were placed in the supine position with their necks extended, and their heads were tilted in the direction opposite of the sonographer^**(19)**^. Parotid and submandibular salivary glands were evaluated by ultrasound to exclude parotitis and to apply other exclusion criteria by using grayscale ultrasonography initially. Elastograms were acquired in the transverse plane of the parotid gland and the longitudinal plane of the submandibular gland to obtain SWE images, following anatomical sites. The transducer was placed on the skin with ample gel and slight contact to minimize artifacts, ensuring no gaps. A Q-Box was positioned over the area where measurements were to be taken, avoiding blood vessels and lymph nodes. When a light yellow color was obtained, indicating that the quality indicator was optimal, SWE measurements were made. For each gland, three equal-sized regions of interest with a diameter of 1 mm were used in order to measure tissue stiffness (in kPa), and the mean value was calculated (Figure 1). To analyze the reliability of the stiffness measurement of the salivary glands, interand intra-rater reliability were assessed by calculating the intraclass correlation coefficient (ICC).

### Statistical analysis

The study data were analyzed with the IBM SPSS Statistics software package, version 22.0 (IBM Corp., Armonk, NY, USA). The normality of data was assessed with the Kolmogorov-Smirnov test. Descriptive statistics, including mean and standard deviation, are reported for continuous variables that exhibited a normal distribution. Interand intra-rater reliability are presented as estimated ICCs and their 95% confidence intervals (CIs). Categorical data are presented as frequency and percentage. To compare normally distributed continuous variables, we used t-tests for independent samples. Qualitative data were analyzed by using the chi-square test. The predictive values were obtained through receiver operating curve (ROC) analysis of the quantitative data. Intragroup comparisons for variables with a normal distribution were made by calculating Pearson’s correlation coefficient. The significance level for all statistical tests was set at 0.05.

## RESULTS

All patients with AS who presented to the rheumatology outpatient clinic during the study period were questioned about sicca symptoms. We identified 87 patients with AS who gave an affirmative answer to at least one of those questions. On blood tests, all of those patients tested negative for antinuclear, anti-Ro, and anti-La antibodies. A total of 21 patients were excluded for presenting with the following: autoimmune thyroiditis (n = 4); diabetes mellitus (n = 4); other rheumatic diseases (n = 3); a history of radiotherapy to the head and neck region (n = 2); a history of anticholinergic drug use (n = 2); and parenchymal changes (heterogeneity, ductal change, or hypoechoic areas) or masses on B-mode ultrasonography (n = 6). Therefore, the final sample comprised 66 patients with AS (the study group) and 71 healthy volunteers (the control group). There was no significant difference between the two groups regarding age or sex (*p* > 0.05).

Parotid and submandibular glands were evaluated with B-mode ultrasonography in the study and control groups. We evaluated the anteroposterior diameters of the glands, parenchymal homogeneity, ductal expansion, the presence of hypoechoic areas, the extent of hypoechoic edema, and the presence of any mass. No significant difference was found between the study and control groups in terms of any of those findings (*p* > 0.05). In both groups, the right and left glands were equal in size and the parenchymal appearance was homogeneous.

For parotid gland SWE, inter-rater reliability was good (ICC: 0.80; 95% CI: 0.52 to 0.99); and intra-rater reliability was good for operator 1 (ICC: 0.85; 95% CI: 0.61 to 1.00) and for operator 2 (ICC: 0.81; 95% CI: 0.65 to 0.99). For submandibular gland SWE, inter-rater reliability was good (ICC: 0.80; 95% CI: 0.50 to 0.99); and intra-rater reliability was good for operator 1 (ICC: 0.88; 95% CI: 0.58 to 1.00) and good for operator 2 (ICC: 0.85; 95% CI: 0.60 to 1.00).

**Figure 2:**
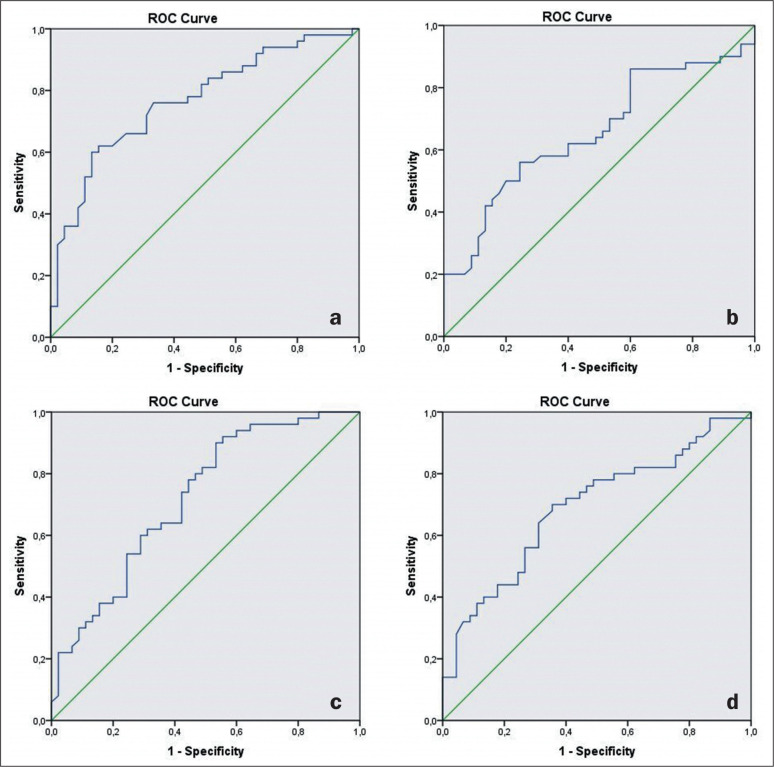
ROC curves plotted for the left and right parotid glands (a and b, respectively), as well as for the left and right submandibular glands (c and d, respectively), to evaluate stiffness by using SWE in patients with AS.

The stiffness values obtained by SWE for the parotid and submandibular glands were found to be significantly higher in the study group than in the control group (*p* < 0.05) (Table 1). Among the patients with AS, the median disease duration was 5 years (interquartile range: 1-16 years). No statistically significant correlation was found between salivary gland stiffness and disease duration (*p* > 0.05). The results of the ROC analysis performed to obtain the predictive values for salivary gland stiffness on SWE in patients with AS are shown in Table 2 and Figure 2.

**Table 1:** Demographic and clinical characteristics of the participants, by group.

Characteristic	Study group (n = 66)	Control group (n = 71)	*P*
Sex, n (%)			0.77^†^
Female	35 (25.6)	36 (26.2)	
Male	31 (22.6)	35 (25.6)	
Age (years), mean±SD	31.98 ± 6.54	28.80 ± 7.48	0.22^‡^
Stiffness (kPa), mean±SD			
Left parotid gland	28.49 ± 5.74	22.85 ± 5.37	0.001^*^,‡
Right parotid gland	27.21 ± 8.37	23.26 ± 5.24	0.008^**^
Left submandibular gland	24.96 ± 6.42	19.75 ± 6.10	0.001^**^
Right submandibular gland	22.92 ± 6.61	18.96 ± 5.14	0.002^**^

* Statistically significant.

† Pearson’s chi-square test.

‡ t-test for independent samples.

**Table 2:** ROC curve analysis of parotid and submandibular gland stiffness.

Gland	Cutoff (kPa)	Sensi-tivity	Speci-ficity	AUC	95% Cl	*P*
Left parotid	> 25.63	76.00	66.70	0.768	0.673-0.863	0.001^*^
Right parotid	> 25.41	62.00	60.00	0.656	0.546-0.766	0.009^*^
Left submandibular	> 20.80	74.00	57.80	0.719	0.617-0.822	0.001^*^
Right submandibular	> 20.12	70.00	64.40	0.690	0.583-0.796	0.002^*^

AUC, area under the curve.

* Statistically significant.

## DISCUSSION

It is well known that AS is a chronic inflammatory disease characterized by axial involvement, which is the prototype of spondyloarthropathies, and that SS is an autoimmune rheumatic disease that generally affects the exocrine glands. As previously stated, SS is categorized as primary or secondary, depending on whether it occurs in isolation or in combination with an autoimmune rheumatic disease. Although the association between SS and autoimmune rheumatic diseases has been frequently investigated and demonstrated, there have been few studies on the combination of SS and AS, and most of those have been in the form of case reports.

As previously mentioned, none of the patients with AS and sicca symptoms in our study sample tested positive for antinuclear, anti-Ro, or anti-La antibodies, and no parenchymal heterogeneity, hypoechoic areas, or ductal changes were detected in the salivary glands evaluated. In the study conducted by Caraba et al.^**(20)**^, demonstrated that neither salivary gland homogeneity nor the presence of only mild changes excludes a diagnosis of SS. However, measurements obtained by SWE showed that parotid and submandibular gland tissue stiffness was greater in the study group than in the control group. This result suggests that there can be patients with AS and sicca symptoms who also have SS and that such patients should be evaluated in more detail. Current SS diagnostic criteria require minor salivary gland biopsy or anti-Ro antibody positivity. However, the methods required are relatively difficult to implement. In patients without antibody positivity but with symptoms, ultrasonographic evaluation of the salivary gland can be an easily applicable and useful technique in clinical practice, whereas biopsy is quite invasive and time-consuming. If there is serious suspicion, patients may be referred for biopsy. In a study investigating whether salivary gland SWE could be included in the diagnostic workup for SS, ultrasonography was found to have lower sensitivity than did biopsy and anti-Ro antibody testing^**(21)**^. However, the authors found that the performance of ultrasonography was comparable to that of the ocular staining score, the Schirmer test, and unstimulated whole saliva flow. In the present study, we aimed to identify an alternative to biopsy and other diagnostic procedures, by including patients who tested negative for antibodies in their blood.

In a study conducted by Kobak et al.^**(4)**^, it was found that 10% of patients with AS also had SS. In another study, involving 105 patients with spondyloarthropathies, Brandt et al.^**(5)**^ found the prevalence of SS to be 7.6% and stated that inflammation in the salivary gland may be caused by common pathogenic mechanisms that have yet to be determined. Di Fazano et al.^**(6)**^ concluded that concomitant SS in female patients with spondyloarthropathies may not be coincidental. The common aspects of those studies is that the patients were diagnosed according to existing criteria, salivary gland evaluation, and antibody testing. Previous studies have also found that SWE is a good, easy-to-apply, noninvasive diagnostic tool for the diagnosis of SS^**(22)**^. The results obtained in the present study, unlike those of other studies evaluating the coexistence of SS and AS, suggest that biopsy, which is an invasive procedure, can be precluded in some cases by emphasizing that biopsy should be performed only in patients with symptoms and with high tissue stiffness values on SWE before biopsy. The combination of SS and AS has been studied, and it has been determined that the risk is increased in this disease^**(4,5)**^, although the mechanism has not been elucidated. We believe that it is ethically inappropriate to perform an invasive procedure when there is no clear scientific basis for it. The patients in our sample were evaluated with the SWE technique. As previously mentioned, SWE is a new, easily applied, low-cost method that has been previously researched in many diseases and has been shown to be important. We believe that the results are valuable and can guide case management in this patient population. However, it should be noted that, as demonstrated in the study conducted by Elbeblawy et al.^**(23)**^, the stiffness of the gland may also change in chronic inflammatory diseases affecting the major salivary gland. Therefore, these findings can be nonspecific and it is necessary to make the clinical correlation.

Our study has some limitations. First, the sicca symptoms were self-reported by the patients, who were not evaluated with tests that are more objective, such as the Schirmer test, sialometry, and sialography. In addition, histopathology results were not available. Therefore, SWE findings could not be compared with the degree of inflammation and fibrosis.

## CONCLUSION

Parotid and submandibular gland tissue stiffness on SWE appears to be significantly greater in patients with AS who exhibit sicca symptoms than in healthy controls. We believe that salivary gland SWE can be used in order to identify patients in whom biopsy is indicated for a definitive diagnosis of SS.
